# Methane Oxidation Potential and Niche Differentiation of Aerobic Methanotrophs in Coastal Mangrove Forest Soils along a 370 Km Long Coastline in Taiwan

**DOI:** 10.1021/acs.est.5c06506

**Published:** 2025-08-08

**Authors:** Yo-Jin Shiau, Ting-Kai Chen, Zhongjun Jia, Chiao-Wen Lin, Chih-Yu Chiu

**Affiliations:** † Department of Bioenvironmental Systems Engineering, 33561 National Taiwan University, Taipei 10617, Taiwan; ‡ Agricultural Net-Zero Carbon Technology and Management Innovation Research Center, National Taiwan University, Taipei 10617, Taiwan; § State Key Laboratory of Black Soils Conservation and Utilization, Northeast Institute of Geography and Agroecology, Chinese Academy of Sciences, Changchun 130102, China; ∥ State Key Laboratory of Soil and Sustainable Agriculture, Institute of Soil Science, Chinese Academy of Sciences, Nanjing 210008, China; ⊥ Department of Marine Environment and Engineering, 34874 National Sun Yat-sen University, Kaohsiung 80424, Taiwan; # Biodiversity Research Center, 38017 Academia Sinica, Taipei 11529, Taiwan

**Keywords:** mangrove forest, methanotroph, DNA stable isotope probing, methane oxidation, next generation sequencing, functional gene, Taiwan

## Abstract

Mangrove forests are key blue carbon ecosystems, but their net sink potential depends on methane oxidation in soils. In this study, DNA-based stable isotope probing was employed to decipher the changes of potentially active methanotrophic communities across five different mangrove forests along a 370 km coastline of Taiwan, with dominating the northern sites (Hsinchu and Miaoli) and dominating the central and southern sites (Changhua, Tainan, and Pingtung). The results showed that the CH_4_ oxidation potentials ranged from 10 to 60 nmol CH_4_ g^–1^ hr^1^ in the mangrove forest soils. The *pmoA* sequencing of fresh soils revealed that unclassified *Methylomonadaceae*-affiliated methanotrophs, *Methylomicrobium-jap-rel*, , and were predominant in forests. In contrast, unclassified *Methylomonadaceae*-affiliated methanotrophs and dominated in forests. Furthermore, *Methylomonadaceae*-affiliated methanotrophs were positively correlated to soil TOC and TN. DNA-SIP analysis further identified unclassified *Methylomonadaceae*-affiliated methanotrophs, , and as potentially active methanotrophs in forests. In contrast, other unclassified *Methylomonadaceae*-affiliated methanotrophs and methanotrophs belonging to the deep-sea-1 and deep-sea-3 clusters were potentially active in forests. Overall, this study revealed distinct biogeographic patterns of methanotrophs in Taiwan’s coastal mangrove ecosystems and highlighted the ecological role of *Methylomonadaceae* in mangrove CH_4_ cycling.

## Graphical abstract

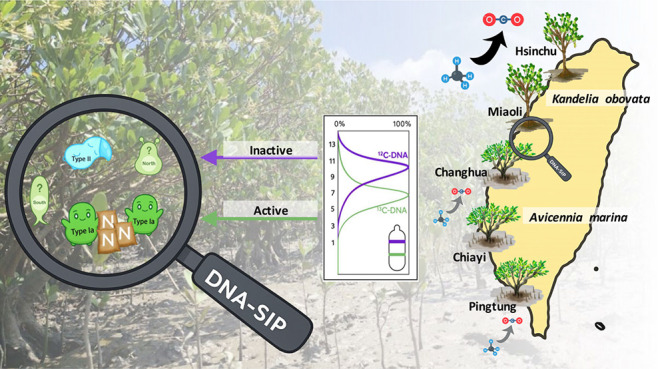

## Introduction

Climate change and global warming are important issues that have been widely discussed in recent decades. Carbon dioxide (CO_2_), methane (CH_4_), and nitrous oxide (N_2_O) are considered major greenhouse gases (GHGs) that can effectively transform solar radiation into heat and warm the atmosphere. With the increase of human activities after the industrial revolution, the concentrations of GHGs are continuing to increase over time and worsen global warming.^1^

Because of the high primary production that many ecosystems, such as forests and wetlands, provide, they are believed to be potential carbon (C) sinks and regulate overall GHG concentrations, especially CO_2_, in the atmosphere. Among all ecosystems, coastal wetlands store high amounts of “blue carbon” and have recently been of high interest to researchers. Indeed, studies have shown that the C storage and C sequestration rates can be high in the soils of coastal wetlands, such as salt marshes and mangrove forests.^2,3^

However, because wetlands provide anaerobic soil conditions, they are also considerable natural GHG sources, especially for CH_4_ and N_2_O, which may largely reduce the net C sequestration effects. Methane is produced in anoxic soil by methanogenic archaea through anaerobic respiration. This process occurs by three major pathways: hydrogenotrophic methanogenesis, acetoclastic methanogenesis, and methylotrophic methanogenesis. The precursors of these pathways, such as acetate and H_2_, are primarily derived from the microbial breakdown of organic matter through fermentation.^4^ However, most microbially generated CH_4_ can be reoxidized when it diffuses to surface soils by a particular microbial community—methanotrophs.^5^ Thus, understanding the activity and composition of methanotrophs and their relation to environmental factors may be beneficial for future coastal wetland management in reducing the overall CH_4_ emission.

Next-generation sequencing (NGS) has enabled comprehensive surveys of microbial diversity in various ecosystems.^6–11^ However, conventional DNA-based approaches, such as metabarcoding and metagenomic sequencing of microbial DNA, cannot distinguish between active and dormant microbial populations, limiting insights into microbial function.^12^ Moreover, certain facultative methanotrophs can utilize alternative C substrates and may not oxidize CH_4_ *in situ*, further complicating the identification of truly active methanotrophs using DNA-based methods.^13–16^

To specify the functionally active microbial communities, metatranscriptomics and stable isotope probing (SIP) have been applied.^17–21^ While metatranscriptomics are powerful, they can be costly^17^ and less efficient for capturing low-abundance functional groups.^12^

DNA-based stable isotope probing (DNA-SIP) offers a more cost-effective alternative for identifying potentially active microbial groups.^22–28^ This approach has been widely used to investigate potentially active methanotrophic communities in various ecosystems.^24,26,27,29–31^ Studies have shown that environmental factors, such as salinity, ammonium (NH_4_^+^), pH, and temperature, play critical roles in shaping methanotrophic communities,^32,33^ highlighting the importance of site-specific analyses.

Unlike freshwater ecosystems, mangrove soils exhibit unique physicochemical properties, including saline conditions and high sulfate (SO_4_^2–^) concentrations in porewater.^34,35^ These conditions significantly influence microbial communities, particularly carbon and nitrogen cycling processes.^34^ As a key blue carbon ecosystem, mangrove wetlands store large amounts of organic carbon, but their organic matter composition and C/N ratio differ from those of terrestrial soils.^36,37^ Previous studies have shown that *Methylomonadaceae*-affiliated methanotrophs are dominant in tidal and coastal mangrove forests, likely due to their adaptability to saline conditions.^38–40^ In contrast, freshwater wetlands, such as rice paddies, are often dominated by *Methylocystaceae*-affiliated methanotrophs, which thrive in environments with lower SO_4_^2–^ levels and different organic carbon sources.^10,30^

Moreover, studies in mangrove forests have demonstrated that active methanotrophic communities are shaped by environmental gradients, such as tidal inundation frequency, salinity, and temperature. One study focusing on different tidal elevations reported that *Methylomonadaceae*- and *Methylococcaceae*-affiliated methanotrophs dominated soils at higher elevations.^40^ A subsequent study conducted along a salinity gradient within the same estuarine system, also dominated by , further showed that and other halotolerant *Methylomonadaceae*-affiliated methanotrophs became more dominant and active in polyhaline soils in winter.^39^

While these findings highlight the role of local environmental conditions in structuring methanotrophic communities, it remains unclear whether such ecological patterns are consistent across geographically distinct mangrove forests and whether mangrove species’ identity also contributes to shaping active methanotrophic compositions.

Thus, in this present study, we investigated the potential active methanotrophs with a DNA-SIP approach across five different mangrove forests, including two main mangrove species found around Taiwan. While seasonal or interannual dynamics could also shape methanotrophic communities, this study was designed as a spatial comparison to capture geographic variation and provide a baseline for future temporal investigations. We also compared the results with the collected physicochemical properties in order to gain a better understanding of the environmental niches of potential active methanotrophic communities in coastal mangrove ecosystems. By examining these variations, we seek to identify consistent ecological trends and provide new insights into the biogeographic distribution and functional roles of methanotrophs in saline wetland environments.

## Materials and Methods

### Site Description and Soil Sampling

A total of five mangrove forests along the west coast of Taiwan were investigated in this study. The distribution of mangrove species along Taiwan’s west coast varies by location. In northern Taiwan, which is at a higher latitude, is the dominant species, while in southern Taiwan, predominates. These mangroves are mostly found around one meter above the sea level, influenced by the twice-daily tides. Despite the difference in species distribution between the north and south, the day and night temperatures in both northern and southern mangrove forests typically range between 25 and 30 and 15–25 °C during the summer and winter, respectively.

The most northern mangrove forest observed in this study is in Hsinchu City, Taiwan (24°54′30.9″N 120°58′19.2″E), where is the dominant mangrove species, while is a minor mangrove species around the forest edge (as shown in Figure S1). Soil samples were collected from the forest (designated as K1).

The second northern mangrove forest is in Miaoli County, Taiwan (24°40′01.9″N 120°50′31.3″E), where is the only mangrove species at the site. Soil samples were collected from the lower elevation area, where the soil was consistently saturated with water (designated as K2).

The third mangrove forest is in Changhua County, Taiwan (23°55′57.3″N 120°18′52.8″E), where is dominant in the forest. Soil samples collected from this site were designated as A1.

The fourth mangrove forest (23°17′40.1″N 120°06′42.3″E) is located in Tainan County, Taiwan, and is the dominant mangrove species. The site in the fourth mangrove forest is designated as A2.

The fifth mangrove forest is located in Pingtung County, Taiwan (22°26′21.5″N 120°29′29.5″E), where is also the dominant mangrove species. One sampling site was identified and designated as A3.

Soil samples were collected within a two-week period during the low tide in August 2018, under consistent climatic conditions across all sites. Three square plots (25 × 25 m) were identified at each site. These three plots were spatially separated and treated as independent biological replicates. Within each plot, 20 soil core samples, spaced about 5 m apart, were collected with a random walk. Briefly, each soil core was collected with a polyvinyl chloride (PVC) tube, 1.5 cm in diameter and 30 cm long. The PVC tube was pushed 20 cm deep directly into the soil using hands, capped with a silicone stopper at the top, and then withdrawn from the soil. All of the soil samples were stored in an ice box and brought back to the laboratory.

After the collected soils were retrieved from the tubes, the top 0–2 cm of the soil layer was carefully sliced and collected with a knife pretreated with 70% ethanol, as this surface depth has been recognized as the most active zone for methane oxidation in mangrove forests in previous studies.^39,40^ The 20 soil cores from each 25 × 25 m plot were then mixed in a plastic sealed bag to form a composite sample. Thus, each site had three independent composite soil samples, one from each plot, which were analyzed separately (*n* = 3 per site). The composite samples were stored at 4 °C for further analyses.

### Soil Physiochemical Analyses

Collected soil samples (*n* = 3 per site) were analyzed for their texture, pH, soil total organic carbon (TOC), total nitrogen (TN), soil labile C (i.e., soluble organic carbon; S_b_OC), and soil labile N (soluble organic N: S_b_ON; NH_4_^+^; nitrate: NO_3_^–^; total dissolved N: TDN). Soil pH was measured from a 1:1 fresh soil to water ratio using a pH meter with a glass electrode (Jenco 6009, Jenco Instruments, San Diego, California, USA). Soil TOC and TN were measured with an elemental analyzer (Fisons NA1500, ThermoQuest Italia, Milan, Italy). Since the soil samples were collected during low tide, pore water was not available at all sites. To ensure consistency across all sampling sites, soil labile C and N were measured with a 2 M KCl extraction method.^41,42^ First, 5 g of soil was weighed from each sample and mixed with 50 mL of KCl solution in a 250 mL glass flask. The mixture was shaken with a horizontal shaker at 150 rpm for an hour. Then, the mixture was transferred to a 100 mL centrifuge tube and centrifugated at 10,000 × *g* for 10 min. The supernatant was analyzed for NH_4_^+^ with an indophenol method,^43^ for NO_3_^–^ with a cadmium reduction method,^44^ and for total dissolved N (TDN) with a persulfate method.^45^ In addition, S_b_ON was calculated by subtracting NO_3_^–^ and NH_4_^+^ from TDN. S_b_OC was measured by using a TOC analyzer (1010, O.I. Analytical, Texas, USA).

In addition, compositions of soil C contents, including acid-hydrolyzable carbon pool I (AHPI-C) (i.e., biodegradable carbon), acid-hydrolyzable carbon pool II (AHPII-C) (i.e., biodegradable carbon with higher molecular weight), and recalcitrant carbon pool (RP-C) (i.e., nonbiodegradable carbon),^46^ were analyzed with an acid-hydrolyzable method.^42,47^

### Methane Oxidation Potential Experiment and DNA-SIP Microcosms

A DNA-SIP experiment was conducted with the collected samples to determine the fast-growing methanotrophs in mangrove soils, as previously described.^23,48,49^

For the DNA-SIP experiment, two incubation bottles were prepared from each composite soil sample: one with the ^12^C-labeled substrate and the other with the ^13^C-labeled substrate. For each bottle, 10 g of soil was weighed out and placed in a 125 mL glass serum vial. The soil used for incubation was collected directly from the field without adjusting its moisture content, meaning it remained near water saturation. The vial was then plugged with a rubber stopper and sealed with an aluminum crimp seal. Three replicates were injected with 3 mL of ^12^CH_4_, and the other three replicates were injected with 3 mL of ^13^CH_4_ so that the headspace of each vial contained about 20,000 ppm of CH_4_. All of the samples were incubated in an incubator at 20 °C for 14 days (LM-570RD, Yihder Technology Co., New Taipei City, Taiwan).

At days 0, 3, 7, 10, and 14, the CH_4_ concentrations in each sample were measured using a flame ionization detector (GC-FID, GC9720, Fuli Instruments, Zhejiang, China). The CH_4_ oxidation potential of each mangrove forest site was determined from the same DNA-SIP incubation vials by applying linear regression to the measured CH_4_ concentrations over time.

After the incubation experiment, about 0.8 g of soil from each replicate was extracted for the genomic DNA using the PowerSoil DNA Isolation Kit (Qiagen, Hilden, Düsseldorf, Germany) based on the manufacturer’s instructions. In addition, the total DNA was also extracted from the collected fresh soils for comparison.

The total DNA extracted from incubated samples was separated into 15 density gradient fractions by an ultracentrifuge (Optima XPN-80, Beckman Coulter, CA, USA) using CsCl-based isopycnic density gradient centrifugation at 190,000 × *g* for 44 h.^22,50^ The buoyant density for each DNA fraction was measured with a digital refractometer (AR200, Reichert Tech., Ametek Inc., NY, USA). In addition, the absolute abundance of *pmoA* genes in fractions 2–14 was analyzed by real-time quantitative polymerase chain reaction (qPCR) with a primer pair (A189F/mb661r) and a qPCR reagent kit (RR420A, SYBR Premix Ex Taq, Takara Bio Inc., Shiga, Japan). The cycling steps of the qPCR analysis of the *pmoA* gene were as follows: 5 min at 95 °C, followed by 40 cycles of 92 °C for 10 s, 55 °C for 30 s, 72 °C for 30 s, and 80 °C for 12 s with acquisitions. Standard curves were generated using serial dilutions of plasmids containing TA-cloned *pmoA* gene fragments, amplified from environmental DNA using the same primer pair as the qPCR assay (A189F/mb661r).

### Metabarcoding Sequence and Methanotrophic Community Identification

The total DNA from the fresh mangrove soils and the DNA from the fraction of ^13^CH_4_ incubated soils with the highest abundance of *pmoA* genes were sequenced to determine their active methanotrophic compositions (Figure S2). The polymerase chain reaction (PCR) technique was applied to amplify the *pmoA* gene fragments with the same primers and cycling conditions but with a different reagent kit (RR902A, Premix Ex Taq, Takara Bio Inc., Shiga, Japan). In addition, soil 16S rRNA genes were amplified with a universal primer pair (515*F*/907R) and a reagent kit (KAPA HiFi HotStart ReadyMix, KAPA Biosystems, MA, USA) with PCR. The *pmoA* and 16S rRNA amplicons were sequenced using Illumina MiSeq (San Diego, CA, USA).

The sequence classification was conducted using Mothur 1.47 based on the software instructions.^51^ The *pmoA* database from Dumont et al.^52^ was used for analysis. Chimeric sequences in the *pmoA* gene were identified using the FunGene Pipeline.^53^ Then, the representative operational taxonomic units (OTUs) were obtained with a cutoff of 80%.^54^ In addition, the RDP database V18 was used for classifying the 16S rRNA sequences. The microbial communities associated with methanotrophs from 16S sequencing were selected for further analysis based on taxonomic classification using the KEGG database. To identify potential anaerobic methanotrophic archaea (ANME), 16S rRNA amplicon sequences were classified using Mothur 1.47 with the SILVA reference database (version 138). Taxa affiliated with known ANME lineages were identified through genus- or family-level classifications of the sequence data set.

### Data Analysis

The differences among soil physicochemical properties in the studied mangrove sites were tested with a one-way analysis of variance (one-way ANOVA). Tukey’s Honest Significant Difference (HSD) analysis was performed subsequent to the identification of differences in each soil physicochemical property with mangrove forests, as indicated by the one-way ANOVA results. The statistical analyses were calculated with JMP version 11.0 (SAS Inc., Cary, NC, USA).

The relative abundances of methanotrophic taxa among sites were compared using the nonparametric Kruskal–Wallis test implemented in the rstatix package 0.7.2.^55^ in R-Studio 3.6.^56^ A Bonferroni correction was applied to adjust for multiple comparisons.

Phylogenetic trees of methanotrophic OTUs in the studied mangrove forests were calculated with MEGA X.^57^ Redundancy analyses (RDAs) were used to determine the relationship between methanotrophs and soil physicochemical properties using the vegan package 2.5–7 ^58^ in R-Studio. To evaluate the associations between the methanotrophic community composition and soil physiochemical characteristics, Mantel tests were conducted using distance matrix comparisons. Bray–Curtis dissimilarity matrices derived from the relative abundance of methanotrophs (based on *pmoA* and 16S rRNA gene sequences) were compared with Euclidean distance matrices constructed from soil environmental variables. In addition, Spearman correlation analysis was applied to explore the relationships among individual soil parameters. All statistical analyses were performed in R version 4.2.2 using the vegan and Hmisc (v5.2-3) packages.

All of the *pmoA* and 16S rRNA gene sequences obtained from MiSeq sequencing have been deposited in the NCBI under BioProject number PRJNA1094686.

## Results

Results of the soil physicochemical properties showed that the TOC and TN contents were the highest in the A3 site, followed by K1, A2, A1, and K2 sites (Table 1). In addition, the soil TOC:TN ratios were higher than 30 in the K2 and A1 mangrove forests, which were all located in central Taiwan. However, the soil TOC:TN ratios were lower than 15 in K1, A2, and A3 sites and in northern and southern Taiwan. The NH_4_^+^ concentrations were found to be highest in the K1 and A2 sites and decreased in the A1, A3, and K2 sites. The NO_3_^–^ concentrations were slightly higher in the K2 and A3 sites and were the lowest in the K1 site. However, S_b_ON was statistically similar among all of the studied mangrove forests. The TDN concentrations were the highest in the K1 site and were the lowest in the K2, A1, and A3 sites. On the other hand, soil S_b_OC was the highest in the A1 site and the lowest in the A2 site.

**Table 1:** Soil Physicochemical Properties in the Studied Mangrove Forest Soils in Taiwan^a^

Site	Salinity (psu)	TOC (%)	TN (%)	TOC/TN	NH_4_^+^-N (mg N kg^–1^ soil)	NO_3_^–^-N (mg N kg^–1^ soil)	S_b_ON (mg N kg^–1^ soil)	TDN (mg N kg^–1^ soil)	S_b_OC (mg C kg^–1^ soil)	S_b_OC/S_b_ON
K1	20	1.9 ± 0.17^b^	0.16 ± 0.01^b^	11.9 ± 1.6	16.9 ± 0.62^a^	0.1 ± 0.02^d^	18.3 ± 14.1^a^	35.3 ± 13.6^a^	32.5 ± 3.7^bc^	2.9 ± 2.6^a^
K2	18	0.98 ± 0.02^d^	0.03 ± 0.05^c^	37.83 ± 0.1	1.8 ± 1.37^b^	0.3 ± 0.02^a^	4.8 ± 4.0^a^	6.9 ± 2.7^b^	35.5 ± 0.9^bc^	13.4 ± 12.4^a^
A1	30	1.05 ± 0.11^d^	N.A.	∞	3.0 ± 2.03^b^	0.3 ± 0.03^b^	6.4 ± 2.3^a^	9.6 ± 2.1^b^	41.0 ± 0.6^a^	7.1 ± 3.1^a^
A2	22	1.50 ± 0.08^c^	0.16 ± 0.00^b^	9.16 ± 0.6	16.6 ± 0.91^a^	0.1 ± 0.02^c^	3.1 ± 2.1^a^	19.8 ± 1.3^ab^	28.8 ± 0.9^c^	13.6 ± 10.3^a^
A3	28	3.35 ± 0.00^a^	0.27 ± 0.02^a^	12.61 ± 0.7	1.8 ± 0.23^b^	0.3 ± 0.05^ab^	12.5 ± 11.2^a^	14.7 ± 11.4^b^	40.0 ± 7.1^b^	5.7 ± 2.6^a^

a N.A.: not available because the TN concentrations were below detectable limits. Values are expressed as mean ± standard deviation (SD). Within each row, means followed by the same lowercase letter are not significantly different at p < 0.05, based on one-way ANOVA and Tukey’s HSD test.

Soil AHPI-C and AHPII-C appeared to be similar (*p* > 0.05) among the studied mangrove forests, except for A3, where the RP-C was higher than in the other mangrove forests (Figure 1 and Table S1). RP-C was the major soil C, which accounted for about 60% of the soil TOC pool. About 20–30% of the soil TOC was comprised of AHPI-C across the studied mangrove forests in Taiwan.

**Figure 1:**
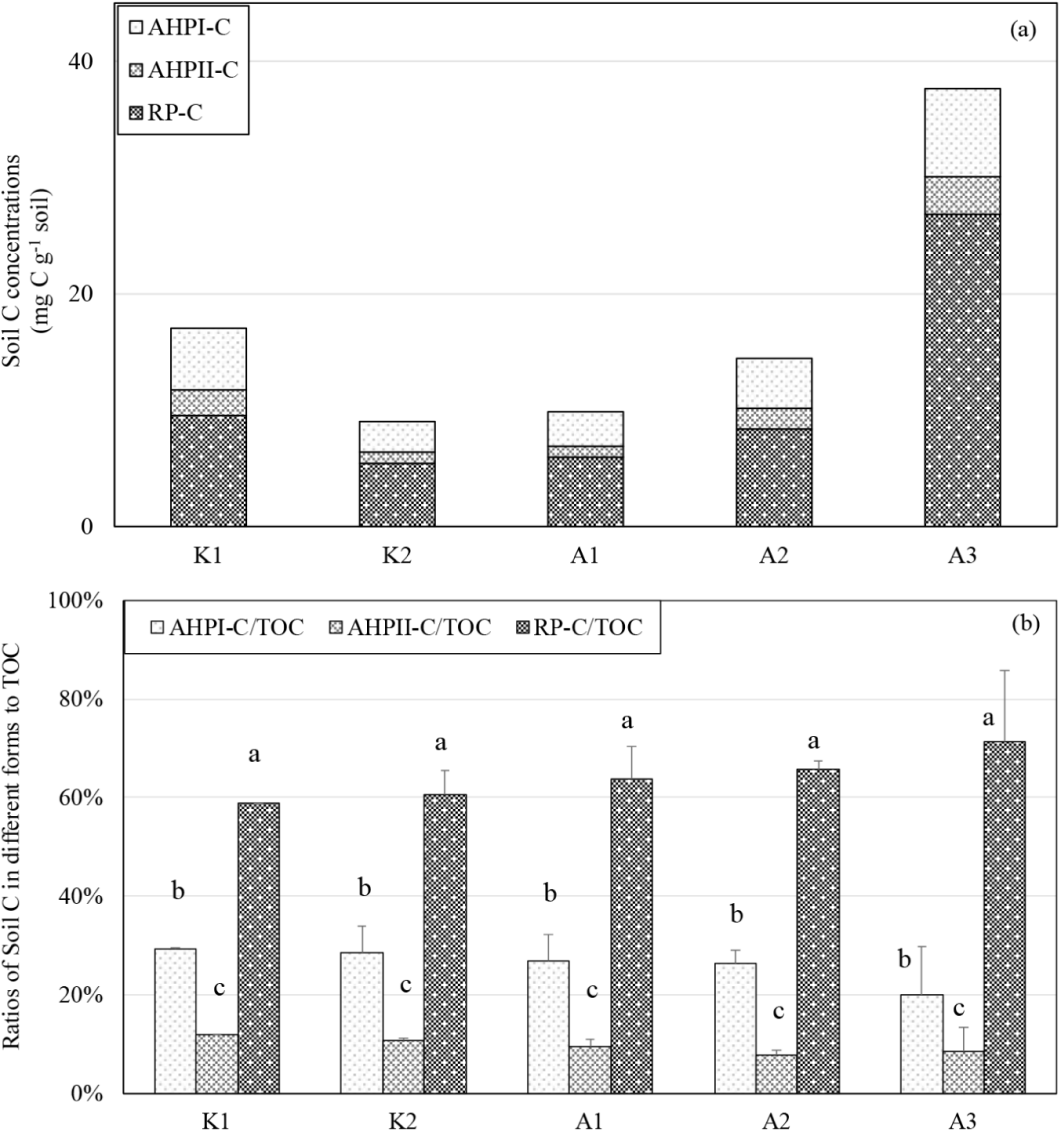
Differentiation of (a) C compositions in different fractions and (b) ratios of soil C individual fractions to total C with acid hydrolysis in the studied mangrove forest soils in Taiwan. AHPI-C: acid-hydrolyzable C pool I (i.e., biodegradable carbohydrate C); AHPII-C: acid-hydrolyzable C pool II (i.e., biodegradable carbohydrate C with higher molecular weight); RP-C: recalcitrant C pool (i.e., nonbiodegradable polyphenol C).^46^ Different lowercase letters indicate significant differences (*p* < 0.05) among C fractions within each site based on Tukey’s HSD test.

The soil CH_4_ oxidation potentials were higher in the (K1 and K2) mangrove forests than those in the (A1, A2, and A3) mangrove forests (Figure 2a). Additionally, statistical analysis using one-way ANOVA showed that the methanotroph population in the fresh soils, based on real-time PCR of *pmoA* genes, was similar among the sites, except for site A1, which showed significantly lower *pmoA* gene abundance (*p* < 0.001) (Figure 2b). Similarly, in the ^13^C-labeled incubation soils, the methanotroph population was comparable among most sites, except for significantly higher abundance at K2 and lower abundance at A2 (*p* = 0.032). A linear regression analysis revealed a significant positive correlation between CH_4_ oxidation rates and log-transformed *pmoA* gene copy numbers in the fresh soils (*p* = 0.043) (Figure S3a), but not for *pmoA* abundances derived from ^13^CH_4_-labeled soils (Figure S3b).

**Figure 2:**
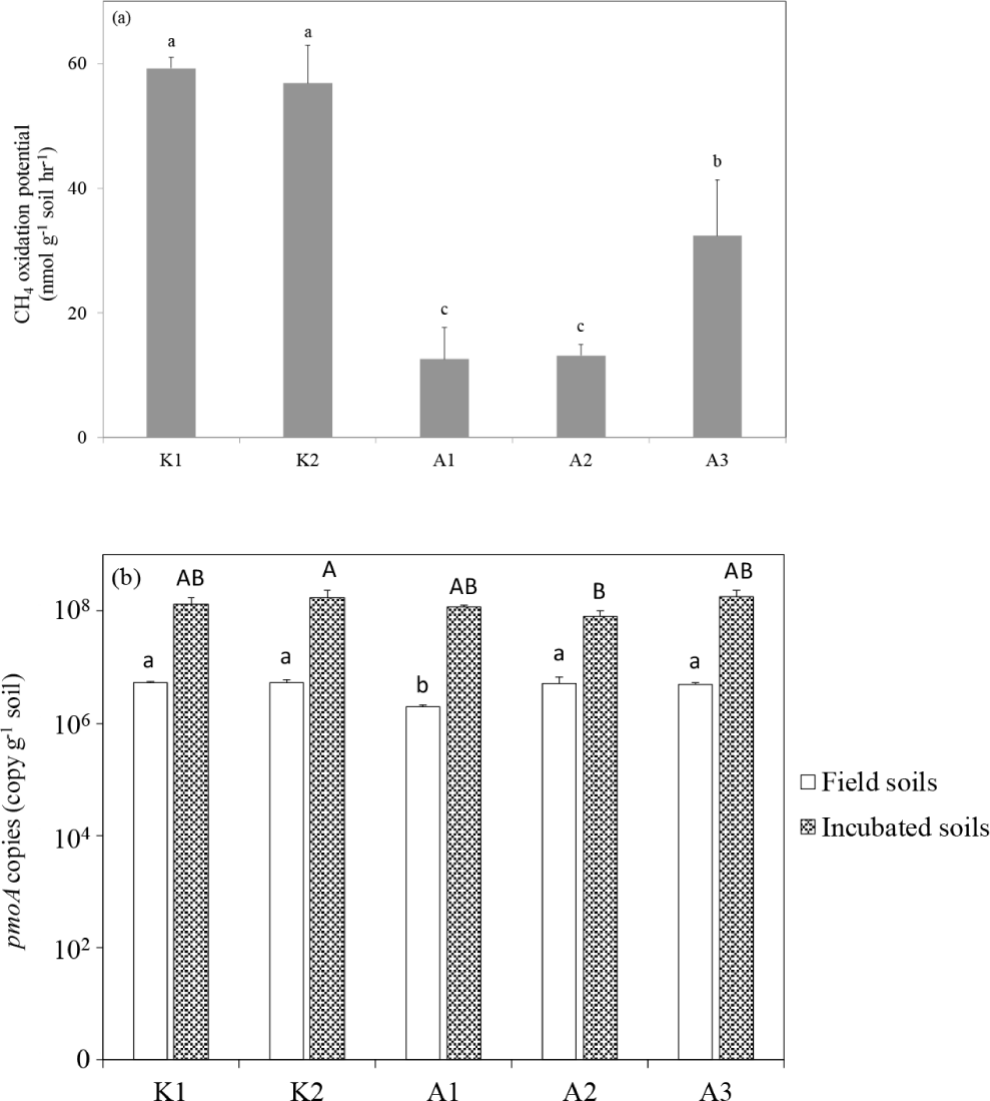
Methane oxidation potential (a) and *pmoA* gene copy numbers (b) in fresh soils and incubated soils (^13^CH_4_ added) from mangrove forest sites in Taiwan. Methane oxidation potential was determined based on the consumption dynamics of 41,000 nmol CH_4_ per 10 g of soil. The headspace CH_4_ concentration was maintained at 2% (v/v) in all microcosms. Fresh soils represent day 0, while incubated soils represent the end of incubation after consuming 41,000 nmol CH_4_ per 10 g of soil. Error bars represent standard deviations (*n* = 3). Different letters indicate significant differences (*p* < 0.05) among variables within each site based on Tukey’s HSD test.

For each soil sample, the number of reads obtained from *pmoA* sequencing ranged from 10,100 to 85,068. Results of *pmoA* sequencing of the mangrove field soils showed that methanotrophs belonging to *Methylomonadaceae* and *Methylococcaceae* families (i.e., Type Ia and Type Ib) were the mostly dominant methane-oxidizing bacteria in the mangrove forest soils around Taiwan, while *Methylomonadaceae*-affiliated methanotrophs appeared to be more widely distributed than *Methylococcaceae*-affiliated methanotrophs in the mangrove forests (Figures 3, S4 and Table S2). The methanotrophs, (*Methylocystaceae* family, Type II), were found with less than 10% of the relative abundance in the studied mangrove forest soils in Taiwan. In addition, a large percentage of unclassified methanotrophs that are distinctively related to *Methylomonadaceae* family (i.e., Type Ia) methanotrophs were observed in the mangrove forests (Figure S4). Similarly, considerable amounts of unclassified methanotrophs (i.e., MOB-like) were found in the studied mangrove forests.

**Figure 3:**
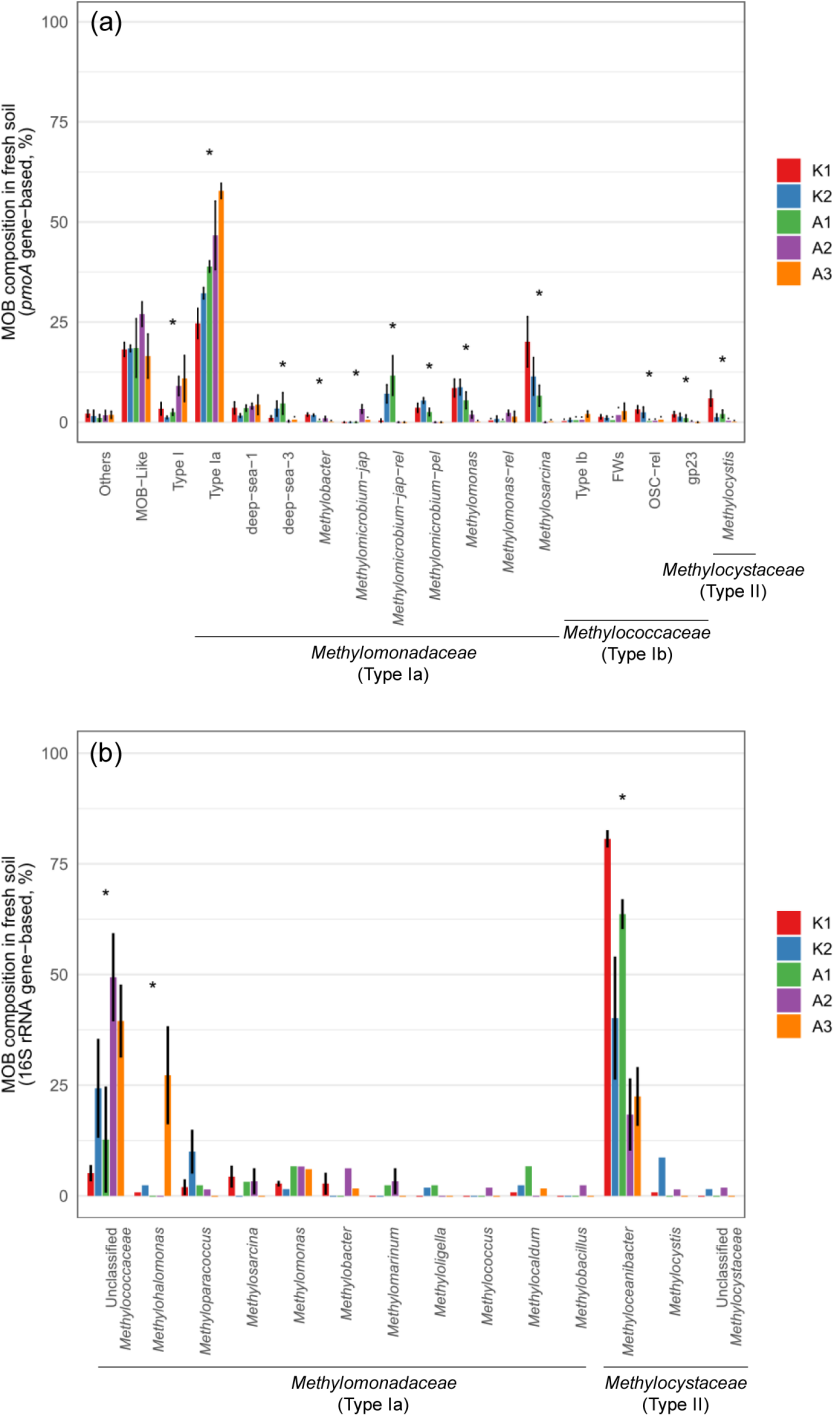
Relative abundance of methanotrophs identified using *pmoA* (a) and 16S rRNA (b) genes in fresh mangrove soils in Taiwan. Taxa with relative abundance <1% were grouped as “Others”. Error bars represent mean ± STD (*n* = 3). Asterisks above bars indicate significant differences in the relative abundance of each taxon among sites based on the Kruskal–Wallis test with Bonferroni correction (*: *p* < 0.05, **: *p* < 0.01, ***: *p* < 0.001).

For 16S rRNA sequencing, the number of reads obtained ranged from 35,478 to 56,796 per soil sample. Results of 16S rRNA analysis, however, showed that methanotrophs belonging to the *Methylocystaceae* family (Type II), such as and , were dominant in most of the studied mangrove forest soils, especially at the K1 site (Figure 3b and Table S2). In addition, unclassified methanotrophs belonging to the *Methylomonadaceae* family were mostly dominant in the A3 mangrove forest soils. Meanwhile, 16S rRNA gene classification also identified ANME, accounting for approximately 6–18% of the total sequences across the studied mangrove soils, except at site A2, where the ANME abundance reached nearly 40%. Among these, , Sh765B-TzT-35, and wb1-A12 were the predominant lineages detected (Figure S5).

Results from RDA of the environmental factors and methanotrophic communities in the field mangrove soils showed that nutrients such as NO_3_^–^, NH_4_^+^ and TDN may favor the genus (Type Ia), while the *Methylococcaceae*-affiliated methanotrophs (Type Ib) and (*Methylocystaceae* family, Type II) were hardly affected by soil C and N (Figure 4).

**Figure 4:**
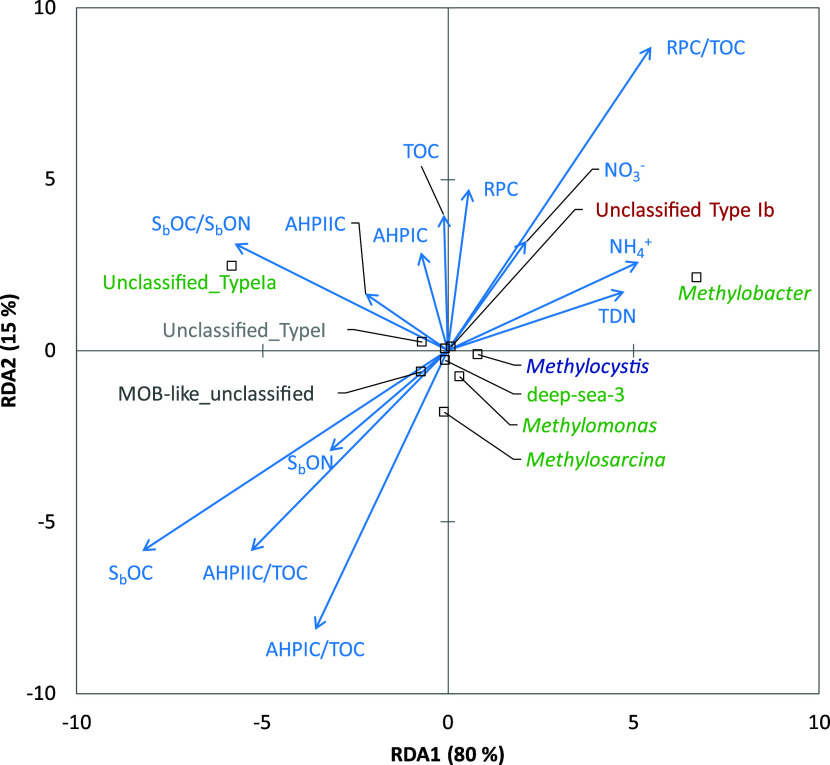
Redundancy analysis (RDA) between methanotrophs identified with *pmoA* genes and physicochemical properties in mangrove forest soils in Taiwan (S_b_OC: soluble organic C; S_b_ON: soluble organic N; NH_4_^+^: ammonium; NO_3_^–^: nitrate; TOC: total organic C; AHPI-C: acid-hydrolyzable carbon pool I; AHPII-C: acid-hydrolyzable carbon pool II; RP-C: recalcitrant carbon pool). Black square dots with green, red, and blue text labels indicate the methanotrophs belonging to the *Methylomonadaceae* family (Type Ia), *Methylococcaceae* family (Type Ib), and *Methylocystaceae* family (Type II), respectively.

Results from the Mantel test revealed significant positive correlations between the methanotrophic compositions and soil physiochemical properties. In particular, the relative abundance of the *Methylomonadaceae* family (Type Ia) was significantly associated with soil NO_3_^–^ and labile organic carbon fractions (AHPI-C and AHPII-C) based on *pmoA* gene analyses (Figure 5a). In addition, *the Methylocystaceae* family (Type II) also showed a positive correlation with the three organic carbon fractions, as well as with soil TOC and TN. Similarly, the *Methylomonadaceae* family (Type Ia) was also positively correlated with the soil nutrients in the 16S rRNA gene-based analyses (Figure 5b), while the *Methylocystaceae* family (Type II) was only positively correlated with TN.

**Figure 5:**
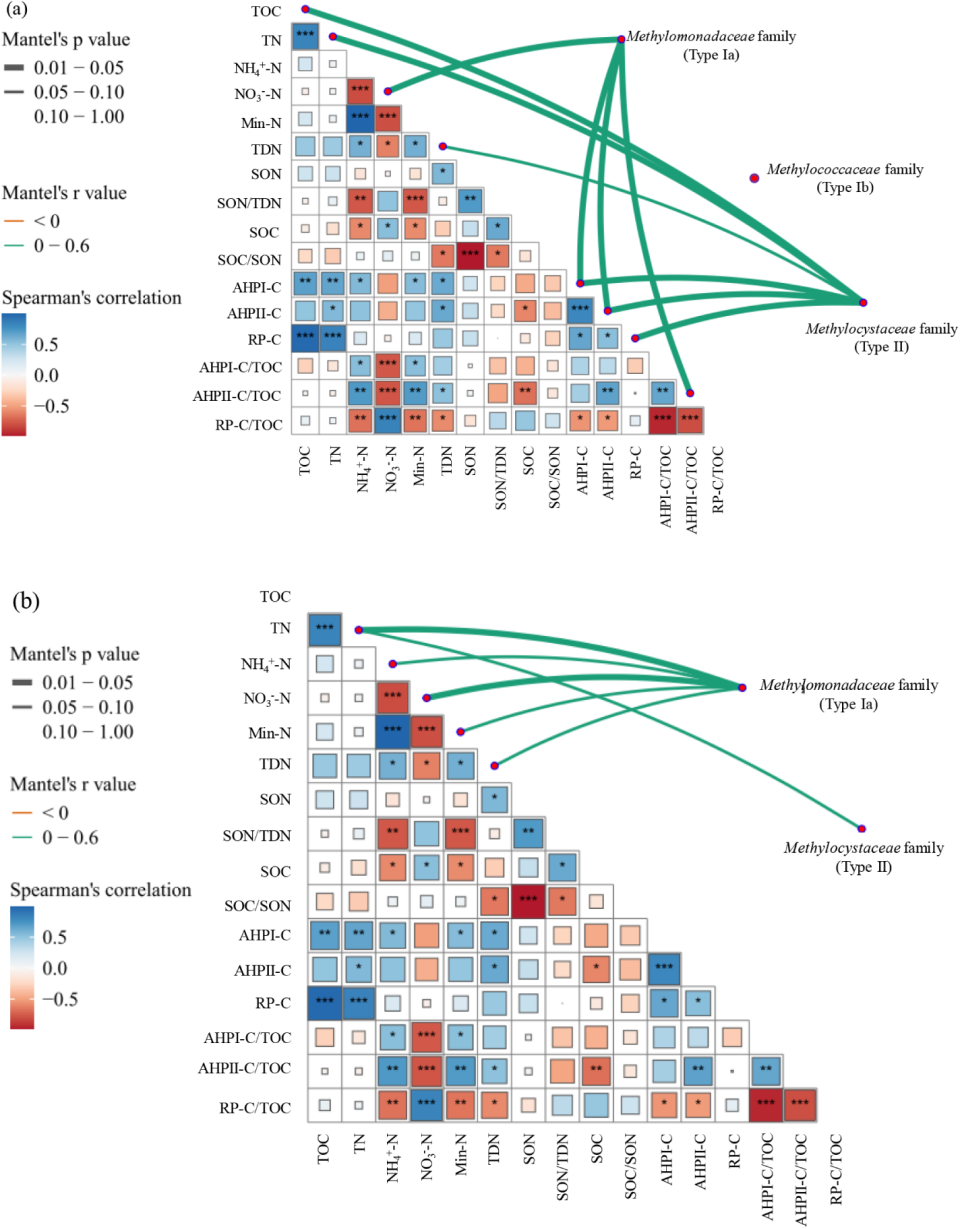
Mantel test results showing the correlations between methanotrophic community composition and soil physicochemical properties in fresh mangrove forest soils of Taiwan based on (a) the *pmoA* gene and (b) 16S rRNA gene sequencing. The strength of Spearman’s correlation between soil variables is presented in the lower triangle, with red and blue color gradients indicating negative and positive correlations, respectively. Asterisks in the matrix indicate the significance levels of Spearman’s correlation, with *p* < 0.05 shown as (*), *p* < 0.01 as (**), and *p* < 0.001 as (***). Connecting lines between methanotrophic families and environmental variables represent Mantel test results, where green lines indicate positive correlations (*r* > 0) and red lines indicate negative correlations (*r* < 0). Line thickness corresponds to statistical significance, with bold lines representing significant correlations (*p* < 0.05) and thin lines indicating marginal correlations (0.05 ≤ *p* < 0.1).

The sequencing results of ^13^C-labeled heavy fraction *pmoA* genes, selected based on *pmoA* abundance profiles and generally corresponding to fractions 5–7 in K1 and fractions 7–9 in the other sites (Figure S2), showed that the methanotrophic communities were spatially varied in the mangrove forest soils after the DNA-SIP incubation. Despite this, all dominant OTUs were affiliated with the *Methylomonadaceae* family (Type Ia) (Figure 6a and Table S3). Potentially active methanotrophs that were distinctively related to , , and methanotrophs belonging to the deep-sea 1 cluster and the deep-sea 3 cluster were found mostly in the mangrove forest soils. On the other hand, potentially active methanotrophs affiliated with unclassified members of the *Methylomonadaceae* family (Type Ia) were predominantly found in the mangrove forest soils.

**Figure 6:**
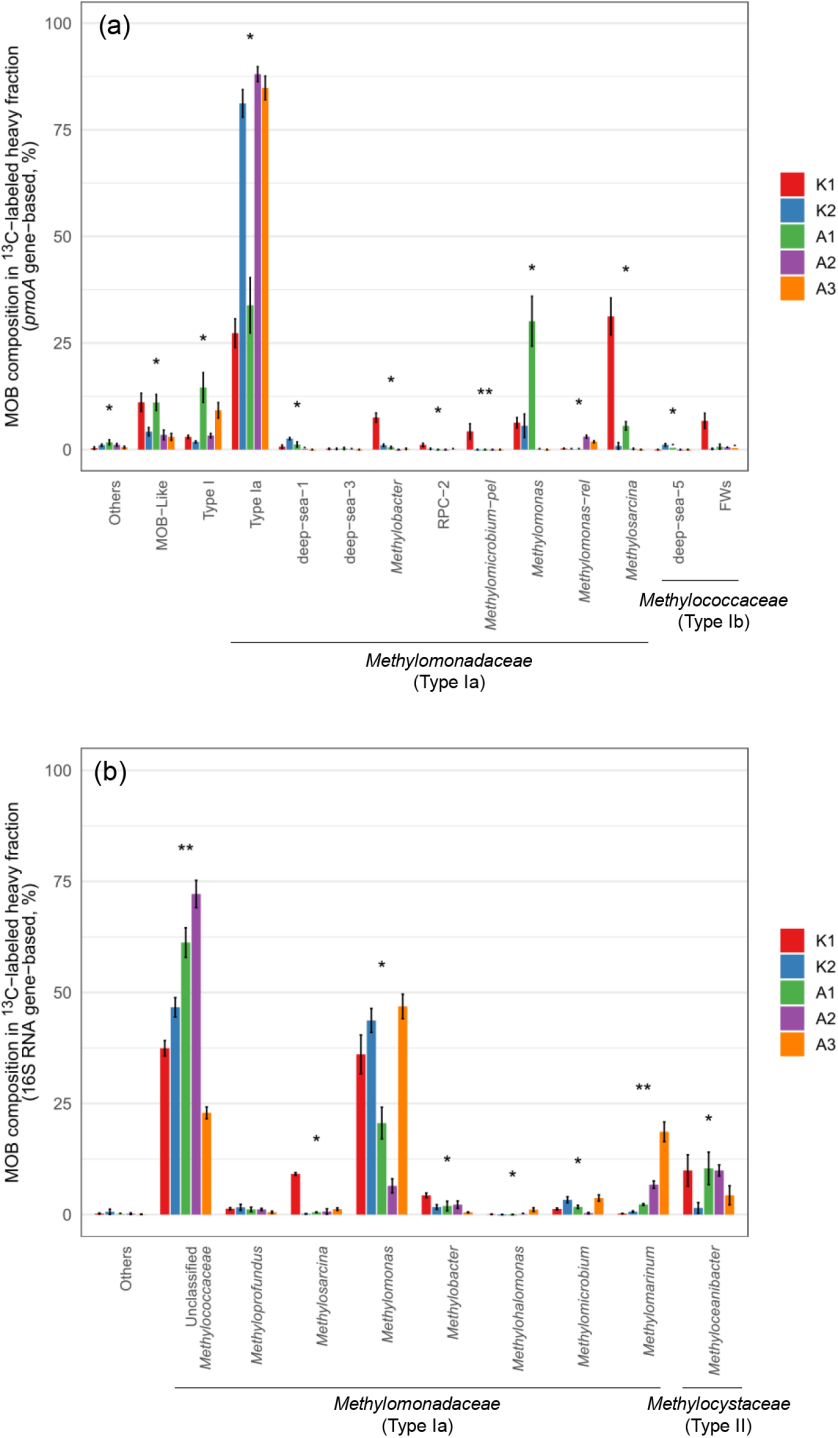
Relative abundance of methanotrophs identified using *pmoA* (a) and 16S rRNA (b) genes in the ^13^C-labeled heavy DNA fraction of mangrove soils in Taiwan. Taxa with relative abundance <1% were grouped as “Others”. Error bars represent mean ± STD (*n* = 3). Asterisks above bars indicate significant differences in the relative abundance of each taxon among sites based on the Kruskal–Wallis test with Bonferroni correction (*: *p* < 0.05, **: *p* < 0.01, ***: *p* < 0.001).

The results of ^13^CH_4_-labeled 16S rRNA showed dominant methanotrophs appeared to be the unclassified methanotrophs belonging to the *Methylomonadaceae* family and in the ^13^C-labeled heavy fractions of the mangrove forest soils (Figure 6b and Table S3). In addition, about 10% of (*Methylocystaceae* family, Type II) was also labeled through the DNA-SIP experiment.

## Discussion

### Changes of Soil Physiochemical Properties among the Mangrove Forest Soils in Taiwan

This study investigated the physiochemical properties and the active aerobic methanotrophs in mangrove forest soils in Taiwan. The results of soil physiochemical property analysis showed that soil TOC concentrations were spatially varied in the mangrove forest soils of Taiwan. Previous studies in mangrove forests showed that the soil TOC contents widely ranged between 0.8 and 2.8% in Asia^59–61^ and 17–34% in North and South America.^62,63^ Soil TOC is fragile, and more than half can be easily lost in anthropogenic or nonanthropogenic disturbances.^63–65^ The low TOC contents observed in most of the mangrove forest soils in Taiwan implied that these sites were mostly under disturbance.^34,66^ Furthermore, because the studied mangrove forests are located in tropical and subtropical zones, they are exposed to warmer air temperatures than the mangroves in other studies and are favorable to litter decomposition.^67–69^ On the other hand, the similar AHPI-C/TOC and AHPII-C/TOC ratios implied that the compositions of soil organic C were not affected by mangrove species or the geographical locations of this study. This observation aligns with previous findings, showing that the composition of soil organic matter (SOM) in mangrove forests was unaffected by species type or site location^64^ and was more influenced by decomposition processes and environmental factors.^70,71^

Similarly, the soil TN and soluble N (i.e., NH_4_^+^, NO_3_^–^ and S_b_ON) varied widely across the mangrove forests in Taiwan. However, these differences of soil N concentrations were not related to the mangrove types but rather exhibited a spatial pattern, with higher concentrations in the northern and southern regions and lower concentrations in central Taiwan (Table 1). While the underlying causes of this pattern remain uncertain, potential contributing factors may include regional differences in environmental conditions or external nitrogen inputs, which warrant further investigation.

### Activity, Composition, and Environmental Adaptation of Methanotrophs in Mangrove Forest Soils

The CH_4_ oxidation potentials analyzed in the mangrove soils were within the ranges of those of other mangrove studies^39,40^ but were higher than those in freshwater ecosystems, such as rice paddies^22,72–74^ measured using similar incubation methods. Similarly, the *pmoA* copies in the fresh and incubated mangrove forest soils were within the ranges of other mangrove ecosystems^39,40^ but were considerably higher than those in freshwater ecosystems.^22,74–76^ This result implies that the mangrove forest environment provides enhanced methanotrophic community growth. In addition, the CH_4_ oxidation potentials were mostly higher in the forest soils than in the forests. The *pmoA* gene copy numbers exhibited modest variation across sites, with the most notable differences observed at site A1 in the fresh soils and at site A2 in the incubated soils.

Further analysis revealed a statistically significant linear relationship between CH_4_ oxidation rates and the log-transformed *pmoA* gene copy numbers in the fresh soils, indicating that even slight differences in methanotroph abundance may still contribute to variations in the CH_4_ oxidation potential. However, the relatively low *R* ^2^ value also suggests that methanotroph abundance alone does not fully explain the observed site-level differences of CH_4_ oxidation potential. Instead, the differences in CH_4_ oxidation rates may be attributed to variations in community composition, where certain methanotrophic species with inherently higher CH_4_ oxidation activity are more prevalent at specific sites.^39,40^ Additionally, environmental factors such as oxygen availability, organic matter composition, salinity, or SO_4_^2–^ concentrations may further influence the selection and relative abundance of these high-activity methanotrophs, thereby affecting overall CH_4_ oxidation rates.^77,78^ Notably, no significant correlation was observed between CH_4_ oxidation potentials and *pmoA* abundances derived from ^13^CH_4_-labeled samples, suggesting that the measured oxidation potentials are primarily linked to methanotrophic populations present in the original field soils rather than those selectively enriched during the SIP incubation.

Because aerobic methanotrophs solely use CH_4_ as the C source,^79^ our results showed no direct relation between TOC and CH_4_ oxidation activity or overall methanotrophic population. However, soil organic C may contribute to CH_4_ production, which, in turn, could influence methanotrophic structure and activity. High CH_4_ concentrations may provide a competitive advantage to certain r-strategy methanotrophs, leading to shifts in community composition despite similar overall population sizes.^5,80,81^ This hypothesis requires further investigation, particularly regarding methanogenic activity in mangrove soils, which could provide more insights into the interaction among soil organic C, CH_4_ production, and methanotrophic dynamics.

In the field of mangrove forest soils across Taiwan, unknown methanotrophs and methanotrophs affiliated with the *Methylomonadaceae* family (Type Ia) dominated 40–80% of the methanotrophic abundance (Figure 3). These observations were consistent with previous studies in coastal ecosystems.^29,39,40,82^ A possible reason for this dominance may be the salinity of the coastal mangrove forest soils. Previous studies observed dominant methanotrophs affiliated with the *Methylomonadaceae* family (Type Ia) in saline lakes and estuaries^29,83,84^ and suspected that these groups may have different physiological responses to high salinity compared to members of the *Methylocystaceae* family (Type II).^32,85^ Furthermore, some studies indicated that and were the most dominant methanotrophs in tolerating high salinity.^29,32,83^ However, the observed dominance of unclassified methanotrophs affiliated with the *Methylomonadaceae* family (Type Ia) and in the present study may imply that methanotrophs other than and also have high resistance to saline environments.

Studies have shown that the *Methylocystaceae*-affiliated methanotrophs are living as k-strategists and have higher adaptation to low CH_4_ concentrations, while the *Methylomonadaceae*- and *Methylococcaceae*-affiliated methanotrophs are r-strategists and have lower metabolic efficiency in CH_4_.^48,86^ This may explain the positive correlation of *Methylomonadaceae*- and *Methylococcaceae*-affiliated (Type I) methanotrophs to soil organic C (i.e., AHPI-C, AHPII-C, RP-C, or TOC), as carbon-rich environments may enhance CH_4_ production in subsurface soils, thereby increasing CH_4_ availability and supporting the growth of these r-strategist methanotrophs.

Notably, although all sites were dominated by *Methylomonadaceae*- and *Methylococcaceae*-affiliated methanotrophs, the relative abundances of and in field soils were substantially higher in K1 and K2 compared to A1, A2, and A3. Moreover, the DNA-SIP results further supported their CH_4_-oxidizing potentials in K1 and K2 soils. These findings suggest that differences in the abundance of high-activity methanotrophs within the same functional group may contribute to the variation in CH_4_ oxidation potential, supporting the notion previously stated that the methanotrophic composition may be more important in determining CH_4_ oxidation activity across mangrove sites than the population size. Future studies incorporating direct measurements of different methanotrophic enzyme activities would be valuable to further validate these observations.

In addition, studies have shown that some *Methylocystaceae*-affiliated (Type II) methanotrophs can acclimate to high NH_4_^+^ environments (i.e., > 70 mM NH_4_^+^)^87,88^ because they can oxidize NH_3_ to hydroxylamine with particulate methane monooxygenase and further detoxify hydroxylamine with hydroxylamine oxidoreductase. However, other studies have demonstrated a higher adaptation of *Methylomonadaceae*-affiliated (Type Ia) (NH_4_^+^: 100 mM; NO_3_^–^: 40 mM) compared to *Methylocystaceae*-affiliated (Type II) (NH_4_^+^: 40 mM; NO_3_^–^: 20 mM) in laboratory with varying NH_4_^+^ and NO_3_^–^ concentrations.^89^ These findings suggest that resistance to NH_4_^+^ and NO_3_^–^ in methanotrophs may occur at the genus of finer levels rather than depending on the types, potentially explaining the inconsistent correlations of *Methylomonadaceae*-affiliated (Type Ia) methanotrophs to soil N nutrients observed in the RDA. In support of these findings, the Mantel test also showed significant correlations of *Methylomonadaceae*-affiliated (Type Ia) methanotrophs with soil nitrogen variables (i.e., NO_3_^–^ and NH_4_^+^).

It is worth noting that all samples in this study were collected within a narrow time window under consistent tidal and climatic conditions to minimize temporal variation, while seasonal shifts in salinity and other environmental parameters may also affect methanotrophic activity and composition, as suggested by previous studies.^39,40^ While the present study focused on geographic and species-level differences, temporal and spatial interactions may exist, and future studies may incorporate both dimensions to further elucidate the ecological controls on methanotrophs in mangrove ecosystems.

In this study, the results from 16S rRNA analysis showed that was one of the dominant methanotrophs in fresh mangrove soils. Previous studies have reported that (i.e., ) oxidizes CH_4_ using only soluble methane monooxygenase (sMMO).^90^ This may be one reason for the difference in the relative abundance of methanotrophs between those with *pmoA* and 16S rRNA gene sequencing results. The *pmoA* gene specifically targets methanotrophs that possess particulate methane monooxygenase (pMMO), whereas 16S rRNA gene profiling captures a broader range of methanotrophs, including those that harbor only sMMO and therefore lack the *pmoA* gene. Consequently, *pmoA*-based analyses may underestimate the abundance of certain sMMO-only methanotrophs, such as . Furthermore, the presence of in the 16S rRNA data set but not in the *pmoA* data set alters the proportional representation of other taxa, contributing to the discrepancy observed between the two approaches.

In addition to aerobic methanotrophs, sequences affiliated with ANME were also detected across all sites, with relative abundances ranging mostly from 6% to 18%, except at site A2, where both ANME-1 and ANME-2 accounted for approximately 40% of the total methanotrophic sequences. Previous studies have shown that ANME-1 lineages often dominate deeper, sulfate-depleted sediment layers, whereas ANME-2 tend to be enriched in sulfate-rich upper layers.^91^ Moreover, NO_3_^–^ reduction to dinitrogen was driven by ANME coupled to sulfate reduction,^92^ while ANME-2d archaea have been shown to perform DNRA and increased NH_4_^+^ production.^93^ ANME-1 groups are also frequently associated with deep marine sediments rich in organic matter.^94^

Although this study did not directly measure SO_4_^2–^ concentrations, site A2 exhibited relatively high NH_4_^+^ concentrations and low NO_3_^–^ and RP-C concentrations, suggesting that the presence of environmental conditions there might be particularly favorable for ANME-1 populations. It is also important to note that our measurements of CH_4_ oxidation potential were conducted under aerobic conditions and thus would not have captured anaerobic CH_4_ oxidation potential by ANME. Future studies may further investigate the ecological roles and spatial distribution of ANME communities in coastal mangrove forests.

### Potential Active Methanotrophs in Mangrove Forest Soils through ^13^C-labeled DNA

The results from the DNA-SIP experiments revealed that the *Methylomonadaceae*-affiliated (Type Ia) methanotrophs appeared to have high CH_4_ oxidizing potential in mangrove forest soils in Taiwan, a finding which was consistent with other studies in different coastal ecosystems.^39,40^ As previously mentioned, many studies have shown that *Methylomonadaceae*- and *Methylococcaceae*-affiliated methanotrophs that have high fecundity and are dominant in carbon-containing nutrients (i.e., r-strategists) can be found in ecosystems with more variable environmental conditions.^48,86^ Some studies claimed that incubation experiments with 10,000 ppm of ^13^CH_4_ might facilitate the growth of *Methylomonadaceae*- and *Methylococcaceae*-affiliated methanotrophs,^48,86^ which may be one reason for the high abundance of active *Methylomonadaceae*-affiliated methanotrophs observed in the present study.

Moreover, most of the potentially active *Methylomonadaceae*-affiliated methanotrophs in the mangrove forest soils are unclassified, especially those in the mangrove forest soils. Although the exact reasons for the spatial differentiation of active methanotrophs are not known, we suspect that organic-rich soils might play a role in affecting the active methanotrophic composition. Phylogenetic analysis of the ^13^C-labeled OTUs from the DNA-SIP experiment revealed that these unclassified taxa formed distinct branches (Figure S4), suggesting that they may represent novel ecotypes adapted to the mangrove soil environment and may play important roles in aerobic CH_4_ oxidation. Future studies may consider isolating these methanotrophs to further elucidate their ecological functions in mangrove ecosystems.

This can also be seen from the RDA results, as unclassified *Methylomonadaceae*- and *Methylococcaceae*-affiliated methanotrophs (unclassified Type I and unclassified Type Ia) were both positively related to the AHPI-C, AHPII-C, RP-C, and TOC in the field mangrove soils, which may reflect the adaptation of microorganisms to specific soil environments, leading to variations in the structural composition of methanotroph communities across different locations.^74^ Nevertheless, the potential active *Methylomonadaceae*-affiliated (Type Ia) methanotrophs in the mangrove forest soils are different from those in freshwater ecosystems, such as rice paddies and lakes.^22,24^ This observation, together with previous studies,^32,39,40^ suggests that *Methylomonadaceae*-affiliated methanotrophs may be better adapted to saline environments.

In addition, although we hypothesized that plant species would influence soil properties, alter CH_4_ oxidation potentials, and shape methanotrophic communities, only higher CH_4_ oxidation potentials were observed in compared to -dominated mangrove forests, without corresponding shifts in measured soil physicochemical parameters or community composition. This may suggest that the influence of vegetation operates through unmeasured variables or microhabitat-scale processes. Furthermore, most of the ^13^C-labeled methanotrophs belonged to the family *Methylomonadaceae* and were composed of diverse, unclassified OTUs (Figure S6), collectively accounting for 50–80% of the total methanotrophic community across sites (Figure 6). The site-specific distribution of these active yet taxonomically unresolved OTUs highlights their potential roles in regulating CH_4_ oxidation and interactions with mangrove vegetation.

The results of 16S sequencing of the heavy fraction of ^13^C-labeled DNA-SIP samples showed that considerable proportions of were active in the mangrove forest soils. Previous studies indicate that many species belonging to are methylotrophs and often coexist with methanotrophs by consuming their effluence.^95,96^ Moreover, studies indicate that methane-oxidizing . can also metabolize other C1–C3 carbon sources and actually prefer carbon sources other than CH_4_.^90^ It is worth noting that has also been reported as a facultative methanotroph capable of utilizing a broad range of multicarbon compounds and may preferentially use alternative carbon sources *in situ*.^13–16^ Given that and were largely detected in the fresh soils but were rarely observed in the ^13^CH_4_-labeled SIP samples, we suspect that most members of this genus may have been utilizing other carbon substrates in situ. Alternatively, the presence of in the DNA-SIP soils may also result from syntrophic interactions with other methanotrophs during the ^13^CH_4_-amended incubation.^95^

## Supplementary Material



## Notes

A map of sampling sites, *pmoA* qPCR profiles across SIP gradients, methane oxidation correlation analyses, a *pmoA* phylogenetic tree, relative abundances of aerobic and anaerobic methanotrophs based on 16S rRNA gene sequences (stacked bar charts), and dominant *pmoA* OTUs in fresh and SIP-labeled soils (heatmaps) (Figures S1–S6) and soil carbon composition and statistical comparisons of methanotroph taxa across sites and treatments (Tables S1–S3) (PDF)
