# Supplementary material for: Methane Oxidation Potential and Niche Differentiation of Aerobic Methanotrophs in Coastal Mangrove Forest Soils along a 370 Km Long Coastline in Taiwan
Source: Environ Sci Technol. 2025 Aug 8;59(32):17022–36. doi: 10.1021/acs.est.5c06506 (PMC12369018; doi:10.1021/acs.est.5c06506)
Supplement: Supplementary file 1 [file es5c06506_si_001.pdf]

## Supporting Information for:

# Methane oxidation potential and niche differentiation of aerobic methanotrophs in coastal mangrove forest soils along a 370 km long coastline in Taiwan

Yo-Jin Shiau[1,2,*], Ting-Kai Chen[1], Zhongjun Jia[3,4,*], Chiao-Wen Lin[5] and Chih-Yu Chiu[6]

[1] Department of Bioenvironmental Systems Engineering, National Taiwan University, Taipei 10617, Taiwan

[2] Agricultural Net-Zero Carbon Technology and Management Innovation Research Center, National Taiwan University, Taipei 10617, Taiwan

[3] State Key Laboratory of Black Soils Conservation and Utilization, Northeast Institute of Geography and Agroecology, Chinese Academy of Sciences, Changchun 130102, China

[4] State Key Laboratory of Soil and Sustainable Agriculture, Institute of Soil Science, Chinese Academy of Sciences, Nanjing 210008, China

[5] Department of Marine Environment and Engineering, National Sun Yat-sen University, Kaohsiung 80424, Taiwan

[6] Biodiversity Research Center, Academia Sinica, Taipei 11529, Taiwan

***corresponding authors:** yshiau@ntu.edu.tw (Y.-J. Shiau); jia@issas.ac.cn (Z. Jia)

**This Supporting Information file includes:**

- **Total pages:** 10
- **Figures:** 6 (Figures S1–S6)
- **Tables:** 3 (Tables S1–S3)

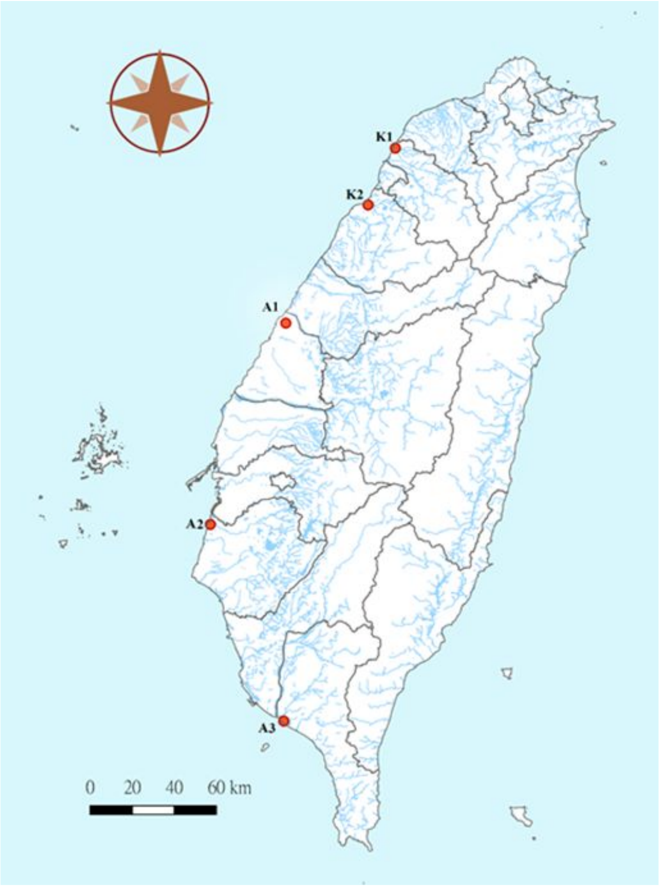


**Figure S1.** The studied mangrove forests in Taiwan (red dots) spanning a 370-km coastline.

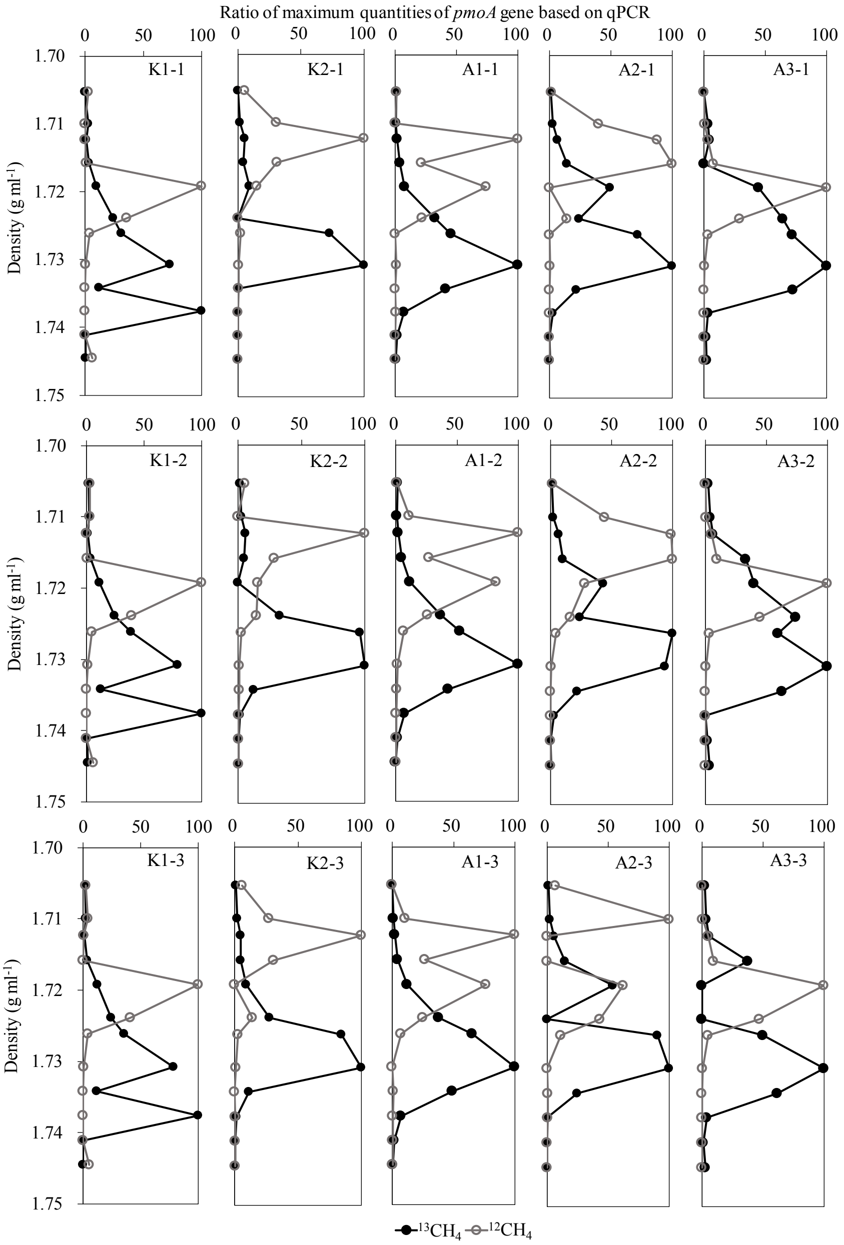


**Figure S2.** Quantitative distribution of methanotrophic *pmoA* gene copy numbers across the entire buoyant density gradient of fractionated DNA samples from five mangrove forest soils in Taiwan, incubated with either $^{13}CH_4$ or $^{12}CH_4$. Each panel shows one of three replicates per site (K1, K2, A1, A2, A3). *pmoA* gene copy numbers were determined by qPCR across density fractions and normalized to the maximum value within each replicate. Solid symbols represent the $^{13}CH_4$-labeled treatments, and open symbols represent the $^{12}CH_4$ controls.

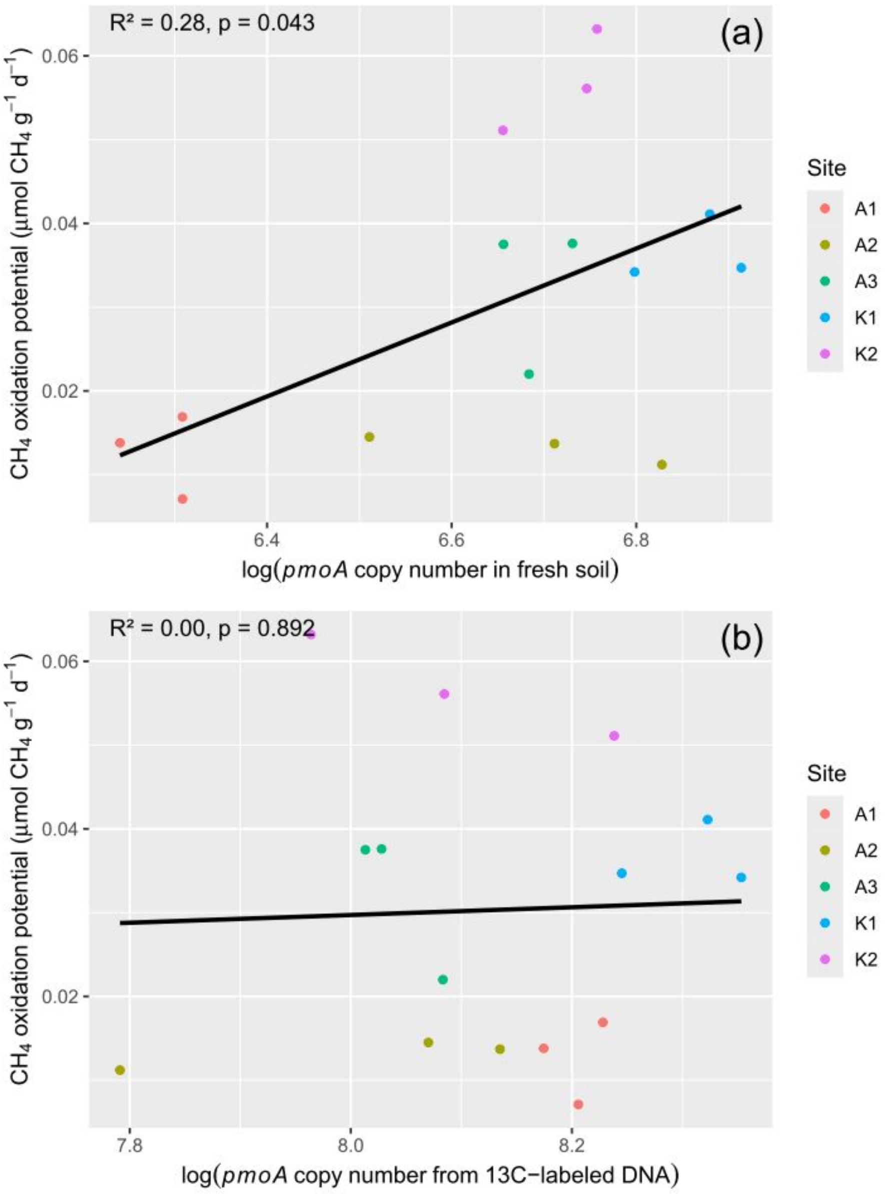


**Figure S3.** Linear regression analyses between $CH_4$ oxidation rates and *pmoA* gene copy numbers in the fresh soils (a), and in the $^{13}C$-labeled soils (b) of the five studied mangrove forests. *pmoA* copy numbers were log-transformed prior to regression analysis. *p* values indicate the statistical significance of the regression slope based on a two-tailed Student's t-test.

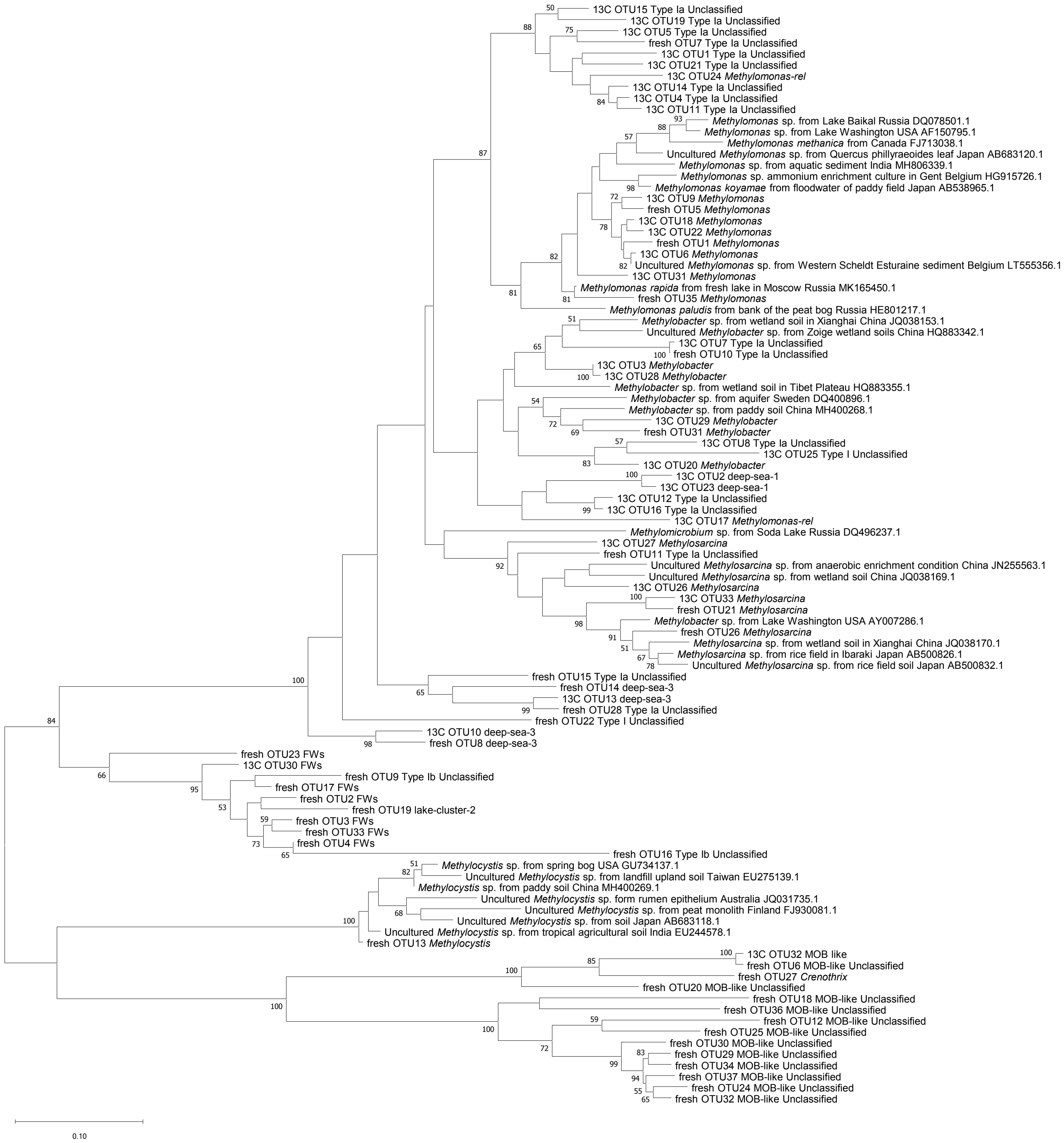


**Figure S4.** The phylogenetic tree of the methanotrophs identified with the *pmoA* genes of fresh and $^{13}C$ labeled mangrove forest soils in Taiwan.

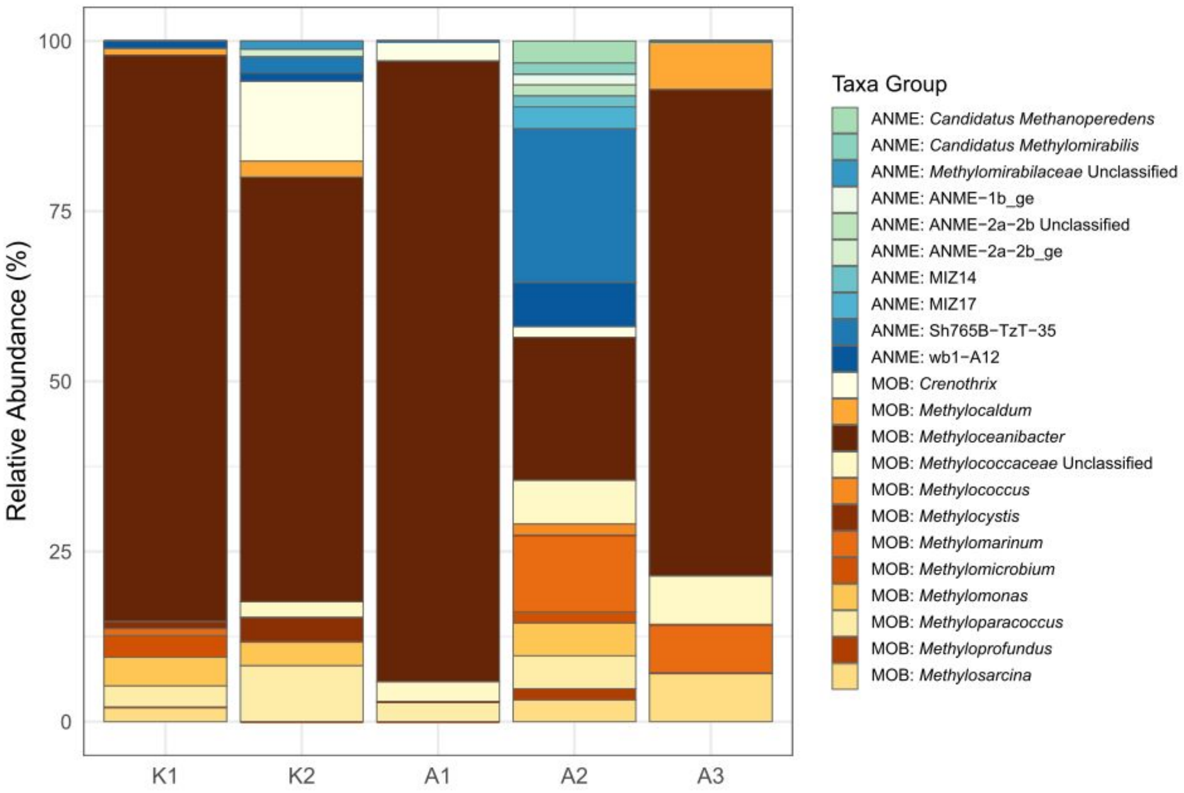


**Figure S5.** Stacked bar chart showing the relative abundances of aerobic (red/yellow) and anaerobic (green/blue) methanotrophs in fresh soil samples collected from mangrove forests in Taiwan. Taxonomic classification was based on 16S rRNA gene sequences using the SILVA 138 reference database.

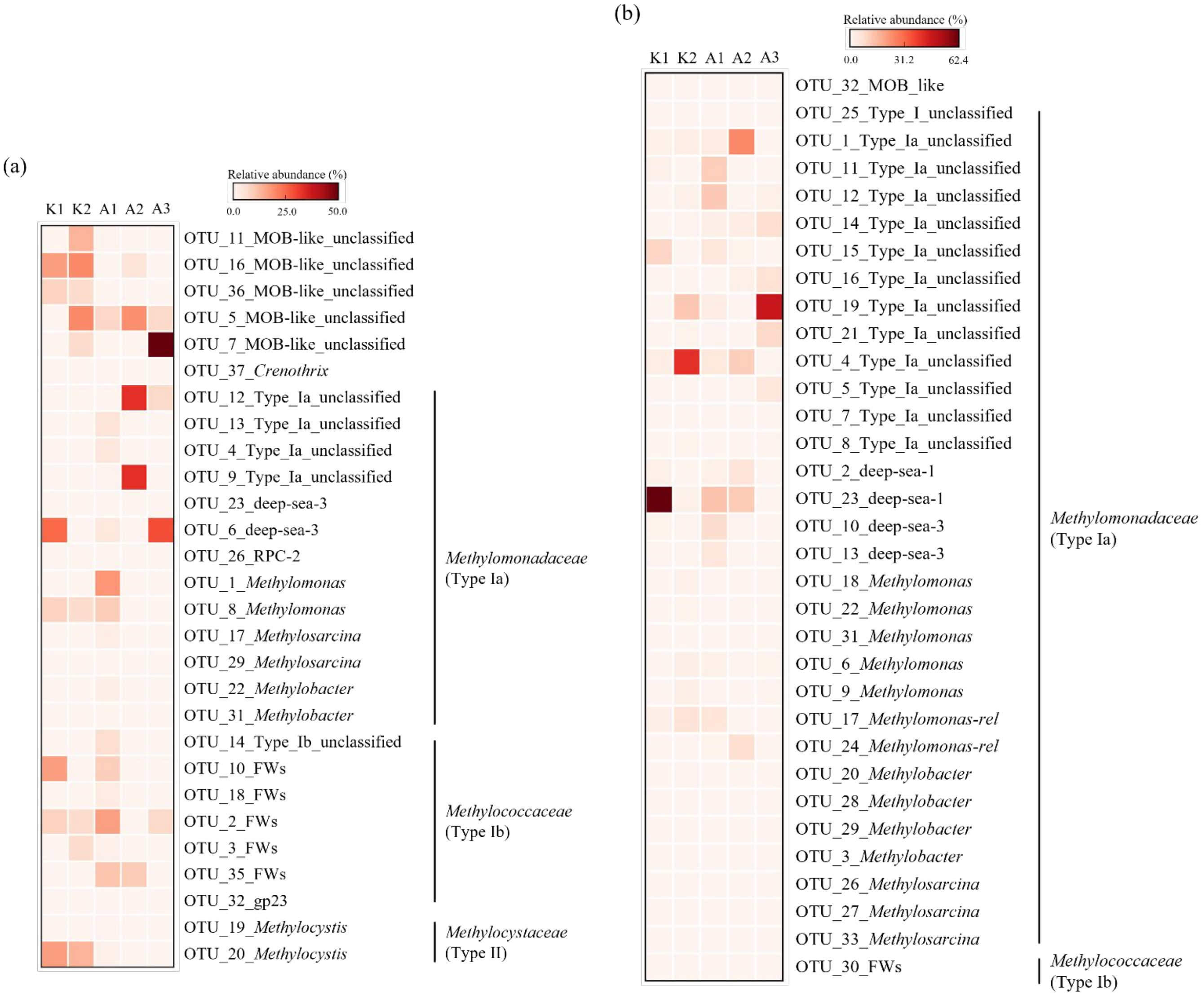


**Figure S6.** Heatmap showing the relative abundance of dominant OTUs derived from *pmoA* gene sequencing in (a) fresh mangrove soils, and (b) the heavy DNA fractions from $^{13}CH_4$-labeled SIP incubations. Only OTUs with ≥ 1% relative abundance in at least one sample are shown.

**Table S1** Differentiation of C compositions in different fraction and ratios of soil C individual fractions to total organic C with acid hydrolysis in the studied mangrove forest soils in Taiwan.

| Site | AHPI-C | AHPII-C | RP-C | AHPI-C/TOC | AHPII-C/TOC | RP-C/TOC |
|---|---|---|---|---|---|---|
| | | (mg C $g^{-1}$ soil) | | | (%) | |
| K1 | 5.65±0.51 | 2.37±0.22 | 11.11±1.02 [b] | 29.55±0.07 | 12.40±0.00 | 58.05±0.07 |
| K2 | 2.32±0.44 | 1.00±0.07 | 6.47±0.65 [b] | 23.75±5.16 | 10.25±0.35 | 66.00±4.81 |
| A1 | 2.68±0.39 | 0.87±0.11 | 6.36±0.97 [b] | 27.20±5.23 | 8.80±1.56 | 64.00±6.79 |
| A2 | 4.47±0.18 | 1.56±0.19 | 8.95±0.77 [b] | 29.35±2.62 | 10.90±0.85 | 59.75±1.77 |
| A3 | 4.51±2.64 | 1.81±1.24 | 26.17±4.87 [a] | 15.8±9.62 | 6.15±4.88 | 78.05±14.50 |

**Notes:** Values are mean ± SD. Different letters indicate significant differences ($p < 0.05$) according to one-way ANOVA and Tukey's HSD post hoc tests. (AHPI-C: acid-hydrolysable carbon pool I; AHPII-C: acid-hydrolysable carbon pool II, and RP-C: recalcitrant carbon pool)

**Table S2** Statistical test results related to significance annotations in the methanotrophic communities in the fresh soils of the five mangrove forests. Differences in relative abundances of each taxon were assessed by Kruskal–Wallis tests followed by Dunn's post-hoc pairwise comparisons with Bonferroni correction.

| Type | Taxon | Statistical Results | *p*-value |
|---|---|---|---|
| *pmoA* | Others | | 0.702 |
| | MOB-Like | | 0.252 |
| | **Type I** | A3= A2 ≥ A1 = K1 ≥ K2 | **0.015** |
| | **Type Ia** | A3 ≥ A2 = A1 = K2 ≥ K1 | **0.011** |
| | deep-sea-1 | | 0.163 |
| | **deep-sea-3** | A1 ≥ K2 = K1 = A3 ≥ A2 | **0.028** |
| | ***Methylobacter*** | * | **0.020** |
| | ***Methylomicrobium-jap*** | * | **0.016** |
| | ***Methylomicrobium-jap-rel*** | * | **0.014** |
| | ***Methylomicrobium-pel*** | K2 ≥ K1 = A1 ≥ A2 = A3 | **0.010** |
| | ***Methylomonas*** | K2 ≥ K1 = A1 = A2 ≥ A3 | **0.017** |
| | *Methylomonas-rel* | | 0.118 |
| | ***Methylosarcina*** | K1 ≥ K2 = A1 = A3 ≥ A2 | **0.012** |
| | Type Ib | | 0.148 |
| | FWs | | 0.320 |
| | **OSC-rel** | * | **0.038** |
| | **gp23** | * | **0.044** |
| | ***Methylocystis*** | K1 ≥ A1 = K2 = A2 ≥ A3 | **0.020** |
| 16S | **Unclassified *Methylococcaceae*** | * | **0.026** |
| | ***Methylohalomonas*** | * | **0.034** |
| | *Methyloparacoccus* | | 0.084 |
| | *Methylosarcina* | | 0.148 |
| | *Methylomonas* | | 0.791 |
| | *Methylobacter* | | 0.277 |
| | *Methylomarinum* | | 0.224 |
| | *Methyloligella* | | 0.519 |
| | *Methylococcus* | | 0.406 |
| | *Methylocaldum* | | 0.849 |
| | *Methylobacillus* | | 0.406 |
| | ***Methyloceanibacter*** | K1 ≥ K2 = A1 = A3 ≥ A2 | **0.013** |
| | *Methylocystis* | | 0.256 |
| | Unclassified *Methylocystaceae* | | 0.519 |

* For some taxa, although Kruskal-Wallis test was significant ($p < 0.05$), Dunn's post-hoc test did not identify significant pairwise differences under Bonferroni correction.

Bolded taxa indicate significance based on Kruskal-Wallis test ($p < 0.05$).

**Table S3** Statistical test results related to significance annotations in the methanotrophic communities in the $^{13}C$-labeled heavy fraction of the five mangrove forests. Differences in relative abundances of each taxon were assessed by Kruskal–Wallis tests followed by Dunn's post-hoc pairwise comparisons with Bonferroni correction.

| Type | Taxon | Statistical Results | *p*-value |
|---|---|---|---|
| *pmoA* | **Others** | * | **0.039** |
| | **MOB-Like** | * | **0.031** |
| | **Type I** | A1 ≥ A3 = A2 = K1 ≥ K2 | **0.012** |
| | **Type Ia** | A2 ≥ A3 = K2 = A1 ≥ K1 | **0.014** |
| | **deep-sea-1** | K2 ≥ K1 = A1 = A2 ≥ A3 | **0.018** |
| | deep-sea-3 | | 0.461 |
| | ***Methylobacter*** | K1 ≥ K2 = A1 = A3 ≥ A2 | **0.012** |
| | **RPC-2** | * | **0.033** |
| | ***Methylomicrobium-pel*** | K1 > K2 = A1 = A2 = A3 | **0.008** |
| | ***Methylomonas*** | A1 ≥ K1 = K2 = A2 ≥ A3 | **0.013** |
| | ***Methylomonas-rel*** | A2 ≥ A3 = K1 ≥ K2 = A1 | **0.011** |
| | ***Methylosarcina*** | K1 ≥ K2 = A1 = A2 ≥ A3 | **0.015** |
| | **deep-sea-5** | * | **0.027** |
| | FWs | | 0.084 |
| 16S | Others | | 0.167 |
| | **Unclassified *Methylococcaceae*** | A2 ≥ A1 = K2 = K1 ≥ A3 | 0.009 |
| | *Methyloprofundus* | | 0.134 |
| | ***Methylosarcina*** | K1 ≥ A3 = A2 = A1 ≥ K2 | 0.028 |
| | ***Methylomonas*** | A3 ≥ K2 = K1 = A1 ≥ A2 | 0.010 |
| | ***Methylobacter*** | K1 ≥ A2 = A1 = K2 ≥ A3 | 0.025 |
| | ***Methylohalomonas*** | * | 0.033 |
| | ***Methylomicrobium*** | A3 ≥ K2 = A1 = K1 ≥ A2 | 0.013 |
| | ***Methylomarinum*** | A3 ≥ A2 = A1 = K2 ≥ K1 | 0.009 |
| | ***Methyloceanibacter*** | * | 0.032 |

* For some taxa, although Kruskal-Wallis test was significant ($p < 0.05$), Dunn's post-hoc test did not identify significant pairwise differences under Bonferroni correction.

Bolded taxa indicate significance based on Kruskal-Wallis test ($p < 0.05$).
